# Treating high-risk moderate acute malnutrition using therapeutic food compared with nutrition counseling (Hi-MAM Study): a cluster-randomized controlled trial

**DOI:** 10.1093/ajcn/nqab137

**Published:** 2021-05-08

**Authors:** Natasha Lelijveld, Claire Godbout, Destiny Krietemeyer, Alyssa Los, Donna Wegner, David T Hendrixson, Robert Bandsma, Aminata Koroma, Mark Manary

**Affiliations:** Centre for Global Child Health, Hospital for Sick Kids, Toronto, Ontario, Canada; Emergency Nutrition Network, Oxford, United Kingdom; Project Peanut Butter, Freetown, Sierra Leone; Washington University School of Medicine, St. Louis, MO, USA; Project Peanut Butter, Freetown, Sierra Leone; Washington University School of Medicine, St. Louis, MO, USA; Project Peanut Butter, Freetown, Sierra Leone; Washington University School of Medicine, St. Louis, MO, USA; Washington University School of Medicine, St. Louis, MO, USA; Washington University School of Medicine, St. Louis, MO, USA; Centre for Global Child Health, Hospital for Sick Kids, Toronto, Ontario, Canada; Ministry of Health, Freetown, Sierra Leone; Project Peanut Butter, Freetown, Sierra Leone; Washington University School of Medicine, St. Louis, MO, USA

**Keywords:** moderate acute malnutrition, wasting, supplementary feeding, RUTF, nutrition counseling, Sierra Leone

## Abstract

**Background:**

There is a lack of consensus on what is the most appropriate treatment of moderate acute malnutrition (MAM).

**Objectives:**

We aimed to determine if provision of ready-to-use-therapeutic food (RUTF) and antibiotics to “high-risk” MAM (HR-MAM) children in addition to nutritional counseling would result in higher recovery and less deterioration than nutrition counseling alone.

**Methods:**

At the 11 intervention clinics, HR-MAM children were given RUTF and amoxicillin along with standard nutrition counseling, for 2–12 wk. All others received 6 wk of nutrition counseling alone. HR-MAM was defined as midupper arm circumference (MUAC) <11.9 cm, weight-for-age *z* score (WAZ) <−3.5, mother not the main caregiver, or a child <2 y old not being breastfed. Outcomes were compared using intention-to-treat analysis.

**Results:**

Analysis included 573 children at the intervention sites and 714 children at the control sites. Of the intervention group, 317 (55%) were classified as HR-MAM. Short-term recovery was greater at the intervention sites [48% compared with 39% at week 12; risk difference (rd): 0.08; 95% CI: 0.03, 0.13]. The intervention group had lower risk of deteriorating to severe acute malnutrition (SAM) (18% compared with 24%; rd: −0.07; 95% CI: −0.11, −0.04), lower risk of dying (1.8% compared with 3.1%; rd: −0.02; 95% CI: −0.03, −0.00), and greater gains in MUAC and weight than did children at the control sites. However, by 24 wk, the risk of SAM was similar between the 2 arms (31% compared with 34%; rd: −0.03; 95% CI: −0.09, 0.02). Control group data identified recent illness, MUAC <12.0 cm, WAZ <−3, dropping anthropometry, age <12 mo, being a twin, and a history of previous SAM as risk factors for deterioration.

**Conclusions:**

Provision of RUTF and antibiotics to HR-MAM children improved short-term recovery and reduced short-term risk of deterioration. However, recovery rates were still suboptimal and differences were not sustained by 6 mo post enrollment.

This trial was registered at clinicaltrials.gov as NCT03647150.

See corresponding editorial on page 835.

## Introduction

Worldwide, wasting affects ∼50 million children each year; however, only 20% of these receive supplementary feeding (1, 2). Wasting encompasses a continuum, including severe acute malnutrition (SAM) and moderate acute malnutrition (MAM). MAM is defined as a weight-for-length *z* score (WLZ) between −2 and −3 SD or a midupper arm circumference (MUAC) between 11.5 and 12.4 cm, without edema (3). Children with MAM are at higher risk of death, disability, infectious illnesses, and deterioration to SAM than healthy children, and those that survive may have lifelong consequences (4). The COVID-19 pandemic has heightened the urgency to reduce wasting and its secondary impacts (5). Whereas there is normative guidance on how best to treat children with SAM (6), there is currently no consistent guidance on how best to manage children with MAM. The WHO has therefore called for more evidence in order to inform feasible and cost-effective guidelines for achieving sustained recovery for children with MAM (7, 8).

Although the difference between MAM and SAM can be as arbitrary as 1 mm in arm circumference, the approaches to treatment differ greatly. SAM is treated with a high-energy ready-to-use therapeutic food (RUTF), whereas MAM is often not treated at all or treated with a variety of approaches including nutrition counseling or 1 of various supplementary food products. Neither counseling nor supplementary feeding programs (SFPs) have demonstrated acceptable recovery rates (9, 10). A recent systematic review found only 11 articles comparing food supplementation with either nutrition counseling, micronutrient supplements, or no treatment, highlighting the paucity of evidence on this topic (10).

There is also growing recognition that treatment should target those at highest risk of death and deterioration (11). Recent trials have aligned the treatment of SAM and MAM by using RUTF as a single food product and suggest this could increase coverage, improve recovery, and simplify supply chains (12–14). However, providing RUTF to all wasted children, both severe and moderate, would be costly and thus difficult to implement at scale.

Our study aimed to inform wasting policy and programs by testing a treatment approach which divides MAM into high- and low-risk populations, and aligns treatment of high-risk MAM with that of SAM. This clinical trial hypothesized that provision of RUTF and amoxicillin to high-risk MAM children in addition to counseling would result in higher recovery and less deterioration than the standard practice of nutrition counseling alone.

## Methods

This was a cluster-randomized controlled clinical trial (NCT03647150) of 22 community nutrition clinics in Pujehun District, Sierra Leone. National data from Sierra Leone estimate that 9% of children <5 y old are wasted (15). The primary outcome was nutritional status 12 wk after enrollment. Each child was categorized as having *1*) recovered from MAM, defined as MUAC ≥12.5 cm; *2*) remained with MAM; *3*) deteriorated to SAM or died; or *4*) been lost to follow-up. Secondary outcomes were reports of recent illness (rash, fever, cough, or diarrhea), weight gain, MUAC gain, subcutaneous body fat, and fat mass distribution (ratio of trunk to peripheral subcutaneous fat). These outcomes were also assessed at 6 and 24 wk after enrollment, including the possibility of relapse in children who had recovered.

A secondary analysis to determine characteristics associated with deterioration (died or developed SAM) was conducted using data from control sites only. The risk factors considered included those used to define high-risk in this study, as well as other anthropometric and demographic characteristics at admission which could plausibly be linked with a poor outcome.

Estimated sample size a priori was ∼800 children with MAM across 20 clusters (clinics). This was adequate for detecting, at 80% power and 5% significance level, a difference in anthropometric recovery rates in the high-risk group from 53% at the control sites to 73% at the intervention sites. This estimation was based on recovery rates for MAM children in Ethiopia who received no support (16) and SFP MAM recovery rates in Sierra Leone. An intracluster correlation coefficient of 0.05 was assumed, a conservative estimate based on the results of a previous cluster-randomized study testing an integrated SAM protocol in Sierra Leone (13).

The 22 clinics were those with the highest attendance in the previous MAM treatment program. The clinics were a minimum of 7 km apart to limit selection bias between participants at each clinic site. The clinic sites were then randomly allocated to either the intervention or the control group, using a spreadsheet-based random number generator (Excel, Microsoft). By design, the intervention was not blinded, because the clinic staff needed to follow the protocol and the caretakers knew whether they received food for their children. The principal investigator and first author were blinded to the allocation group until the point of primary data analysis, in order to maintain objectivity in data management.

### Ethical approval

This study was approved by the following research ethics boards: Sierra Leone Ethics and Scientific Review Committee, Freetown, Sierra Leone; Washington University Human Research Protection Office, St. Louis, USA (reference #201807153); and The Hospital for Sick Kids Research Ethics Board, Toronto, Canada (reference #1000062042).

### Participants

All children aged 6–59 mo with uncomplicated MAM were eligible for enrollment at study clinics. Uncomplicated MAM was defined as MUAC ≥11.5 and <12.5 cm without edema or clinical complications (dehydration, respiratory distress, altered mental status, fever, or a visible sign of developmental delay). Children were screened from those presenting at biweekly nutrition clinics, many of which were referred to the clinic by community health workers doing MUAC screening; self-referral was also possible. Children who were identified as having SAM based on MUAC <11.5 cm or WLZ <−3 at baseline were excluded and treated outside of the study. Any that developed SAM while in the study were referred for SAM treatment but still followed to assess later outcomes. Children were excluded if they were involved in another research trial or feeding program or their caretakers reported an allergy to peanut or milk. Children who defaulted on treatment or missed follow-up visits were still eligible for inclusion at later follow-ups. Caregivers provided written and oral informed consent; the decision to participate in the research did not affect the care received by the child.

### Procedures

At the intervention sites, enrolled children were classified as high-risk (HR-MAM) or low-risk MAM (LR-MAM). Criteria for defining HR-MAM were derived from characteristics associated with failed treatment in MAM SFPs in Sierra Leone (16–18). HR-MAM was defined as having ≥1 of the following criteria: MUAC <11.9 cm, weight-for-age *z* score (WAZ) <−3.5, mother not the primary caregiver, or a child under the age of 2 y not being breastfed (17). At intervention sites, children classified as HR-MAM were provided with 1 daily packet of RUTF (92 g, 520 kcal) and a 7-d course of amoxicillin (40–45 mg/kg per dose twice daily) (19, 20). RUTF was provided until an MUAC >12.4 cm was achieved. All children (at both control and intervention sites) were offered nutrition counseling via mother support groups.

At all clinic visits, weight, length, MUAC, and skinfold thickness were measured using standard WHO clinical procedures (21), including double measurement of MUAC and skinfold thickness, and triple measurement of length. SECA digital weighing scales were used (SECA Ltd.). To assess trunk body fat and fat distribution, skinfold thickness was measured at the subscapular and triceps positions using Tanner/Whitehouse calipers (Holtain Ltd.). Food insecurity was assessed using the Food Insecurity Experience Scale (22).

Children attended clinic fortnightly until treatment was completed and then returned for follow-up at 12 and 24 wk after enrollment. Caretakers participated in biweekly mother support groups delivered by a community respected elder for a total of 4 sessions using a curriculum adapted from the Ministry of Health and Sanitation. This curriculum included optimizing infant and young child feeding, a cooking demonstration, lessons on hygiene and sanitation, health care seeking, child development (UNICEF), and training on MUAC for mothers (ALIMA). The mother support groups are an established program which have shown a 5% increase in recovery from acute malnutrition (13). All children were assessed at the biweekly mother support group meetings to detect anthropometric deterioration or acute illness. If a child was found to have SAM, they were provided with RUTF. A questionnaire was administered to a random 10% subset of participants to ascertain attendance, opinions on the counseling intervention, and compliance to the RUTF dose where applicable. Basic cost differences between the treatment arms were also considered. Further detail on the treatment procedures can be found in the study protocol (17).

### Statistical analysis

Data were double-entered into secure computer databases from paper forms. WLZ, WAZ, and length-for-age (LAZ) *z* scores were calculated using WHO 2006 growth standards with Stata's zscore06 package (23, 24). Analysis of outcomes was conducted at the individual level using a modified intention-to-treat analysis, with adjustment for clustering by clinic. Children were only excluded from analysis if *1*) they did not meet eligibility criteria, *2*) they changed their clinic location and thus allocation group, *3*) their risk category was incorrectly assigned, or *4*) their data card was lost. No imputation of missing data was made. Weight gain was calculated as grams per kilogram per day. Logistic regression analyses were conducted to estimate risk differences for categorical outcomes and linear regression analyses for mean differences for continuous outcomes between study arms. Logistic regression was used to calculate the OR for potential characteristics associated with deterioration during treatment in the control group only. Adjusted analyses included age and sex in the model, selected a priori; all analyses accounted for clustering by clinic site. Venn diagrams were also used to explore the sensitivity and specificity of different combinations of risk factors for detecting high-risk children at admission. Analysis was conducted using Stata IC version 13.1 (StataCorp LP, 2013).

## Results

**FIGURE 1:**
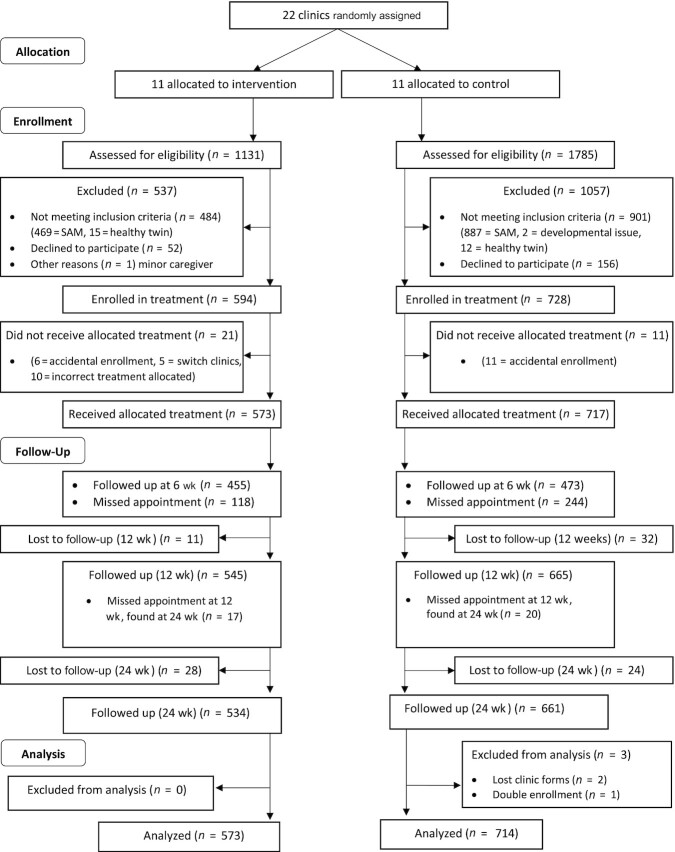
Recruitment flow diagram. SAM, severe acute malnutrition.

A total of 1322 children met the eligibility criteria and were enrolled (**Figure 1**) between November 2018 and December 2019. Of these, 35 children were excluded from analyses. Analyses included 22 clusters and 1287 children at baseline, of which 573 were enrolled at intervention sites and 714 at control; 317 (55%) of the intervention group and 393 (55%) of the control group were classified as HR-MAM (**Supplemental Table 1**). By 12 wk, 43 (3%) children were lost to follow-up; by 24 wk, a further 52 (4%) were lost (Figure 1).

Baseline characteristics were similar in the 2 study arms (**Table 1**, **Supplemental Table 2**), although more children were excluded for presenting with SAM at control clinics than at intervention clinics (Figure 1). Boys were more likely to be classified as HR-MAM than girls owing to greater stunting among boys and therefore a lower WAZ. Among those enrolled, 30.8% reported having SAM previously. Recent morbidity (defined as fever, diarrhea, rash, or cough in the previous 2 wk) was common in all groups (43%).

**TABLE 1:** Baseline characteristics^1^

	Intervention (*n* = 11 sites, *n* = 573)			Control (*n* = 11 sites, *n* = 714)		
	All	High risk (*n* = 317; 55.3%)	Low risk (*n* = 256; 44.7%)	All	High risk (*n* = 393; 55.0%)	Low risk (*n* = 321; 45.0%)
Age and sex						
Males	241 (42.1)	141 (44.5)	100 (39.1)	284 (39.8)	169 (42.9)	115 (35.9)
Age, mo	11 [8–17]	12 [8–19]	11 [8–14]	12 [8–17]	14 [9–19]	11 [8–14]
Older than 24 mo	57 (10.0)	40 (12.6)	17 (6.6)	55 (7.7)	41 (10.4)	14 (4.4)
Anthropometry						
Weight, kg	6.77 ± 0.92	6.74 ± 1.01	6.79 ± 0.79	6.77 ± 0.88	6.77 ± 0.94	6.75 ± 0.80
Length, cm	68.24 ± 5.60	68.57 ± 5.96	67.83 ± 5.09	68.37 ± 5.41	68.83 ± 5.61	67.81 ± 5.09
MUAC, cm	11.94 ± 0.27	11.78 ± 0.23	12.13 ± 0.15	11.97 ± 0.27	11.82 ± 0.25	12.15 ± 0.16
WLZ	−1.73 ± 0.62	−1.88 ± 0.59	−1.54 ± 0.61	−1.76 ± 0.68	−1.91 ± 0.68	−1.58 ± 0.64
LAZ	−2.84 ± 1.16	−3.05 ± 1.25	−2.57 ± 0.97	−2.80 ± 1.18	−3.11 ± 1.27	−2.43 ± 0.92
WAZ	−2.86 ± 0.74	−3.08 ± 0.76	−2.58 ± 0.60	−2.85 ± 0.73	−3.11 ± 0.74	−2.53 ± 0.56
WaSt	150 (26.4)	111 (35.1)	39 (15.4)	206 (28.9)	158 (40.1)	48 (15.1)
Family and environment characteristics						
<2 y and not breastfeeding	63 (11.0)	63 (19.9)	0	93 (13.0)	93 (23.6)	0
Mother not caregiver	49 (8.6)	49 (15.5)	0	65 (9.1)	65 (16.5)	0
Twin	22 (3.8)	17 (5.4)	5 (2.0)	17 (2.4)	8 (2.0)	9 (2.8)
Food Insecurity Experience Scale						
Least insecure (score: 0)	27 (4.8)	17 (5.4)	10 (3.9)	43 (6.1)	28 (7.2)	15 (4.7)
Most insecure (score: 8)	126 (22.2)	60 (19.1)	66 (25.9)	149 (21.1)	71 (18.3)	78 (24.6)
Animals sleep in house	93 (16.2)	46 (14.5)	47 (18.4)	127 (17.9)	75 (19.1)	52 (16.4)
Health						
Reported morbidity in past 2 wk						
Any	248 (43.3)	136 (42.9)	112 (43.8)	323 (45.2)	177 (44.9)	146 (45.6)
Fever	206 (36.0)	113 (35.6)	93 (36.3)	266 (37.3)	146 (37.1)	120 (37.5)
Diarrhea	49 (8.6)	29 (9.2)	20 (7.8)	77 (10.8)	46 (11.7)	31 (9.7)
Cough	119 (20.8)	62 (19.6)	57 (22.2)	164 (23.0)	84 (21.3)	80 (25.0)
Rash	23 (4.0)	14 (4.4)	9 (3.5)	32 (4.5)	17 (4.3)	15 (4.7)
Child ever treated for SAM	182 (31.8)	100 (31.6)	82 (32.0)	215 (30.2)	139 (35.3)	76 (23.9)
Child ever admitted to hospital	77 (13.4)	41 (12.9)	36 (14.1)	78 (11.0)	46 (11.7)	32 (10.1)

^1^Values are mean ± SD, median [IQR], or *n* (%). LAZ, length-for-age *z* score; MUAC, midupper arm circumference; SAM, severe acute malnutrition; WaSt, concurrent wasting and stunting; WAZ, weight-for-age *z* score; WLZ, weight-for-length *z* score.

In a subset of participants surveyed, we found that 58% (72 of 125) attended all 4 mother support group sessions [82% (27 of 33) in the HR-MAM intervention group, 54% (15 of 28) in the LR-MAM intervention group, and 47% (30 of 64) in the control group]. The majority only missed 1 session; only 12% (15 of 125) missed >1 session. Of the 33 HR-MAM caregivers interviewed, all reported that their child enjoyed the RUTF and only 6% (2 of 33) reported that the ration was difficult to finish.

At the end of the treatment, 8 of 1287 (0.6%) children had died and 92 of 1287 (7%) had defaulted on treatment (i.e., missed 3 consecutive visits). Children receiving food were less likely to default (4%) than those receiving only counseling (8%) (*P* = 0.007). In the intervention arm, 38 of 317 (12%) children receiving RUTF remained with MAM after 12 wk of treatment; mean ± SD length of RUTF treatment was 5.4 ± 2.9 wk (range: 2–12 wk).

**FIGURE 2:**
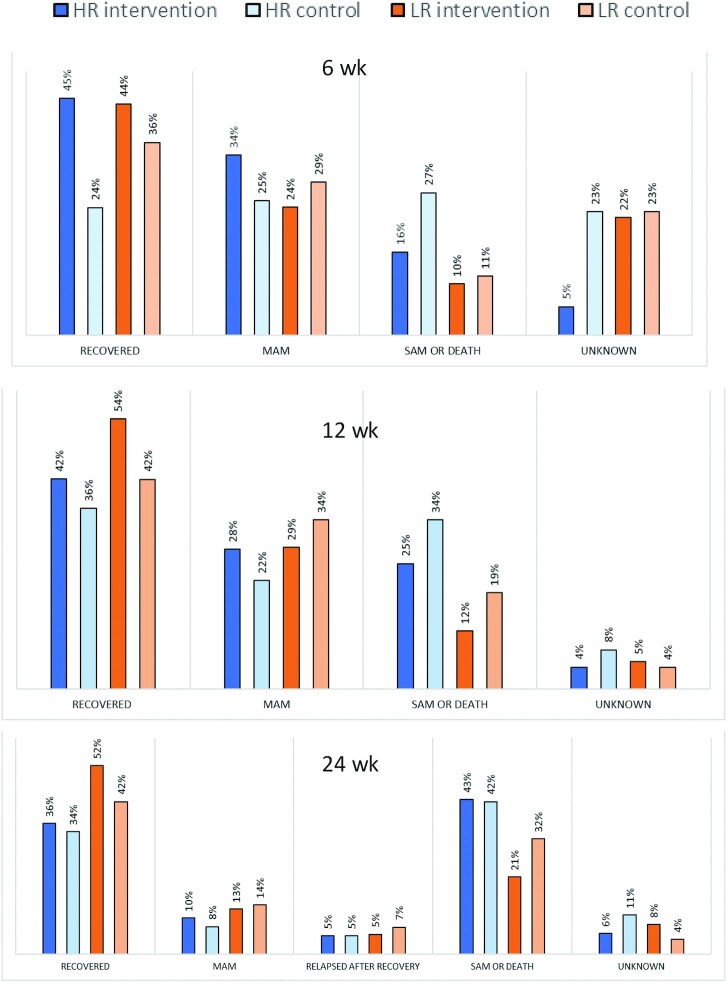
Outcomes at 6, 12, and 24 wk post enrollment. *n* = 317, HR intervention; *n* = 393, HR control; *n* = 256, LR intervention; *n* = 321, LR control. HR, high-risk; LR, low-risk; MAM, moderate acute malnutrition; SAM, severe acute malnutrition.

Recovery was greater for the intervention children at 12 wk than for the control children (48% compared with 39%, *P* < 0.001) (**Table 2**) (disaggregation by HR and LR groups in **Figure 2**). The intervention group had a lower risk of deteriorating to SAM by 12 wk post enrollment (18% compared with 24%, *P* < 0.001) and lower risk of death (1.8% compared with 3.1%, *P* = 0.04) (**Supplemental Figure 1**), and had a greater change in MUAC and average daily weight gain, than did the controls (**Table 3**). These weight and MUAC gains were sustained through 24 wk post enrollment (Table 3). However, by 24 wk, the risk of having deteriorated to SAM was similar between the 2 arms (31% compared with 34%, *P* = 0.24). There was also no difference in risk of relapse by 24 wk for those who recovered (5.2% compared with 6.2%, *P* = 0.46). All groups gained in MUAC between enrollment and the 24-wk follow-up, including the LR-MAM groups who only received mother support group counseling (**Supplemental Figure 2**). The lowest MUAC gains were observed in HR-MAM children at the control sites, among whom 40% deteriorated to SAM and therefore received RUTF. There was no improvement in LAZ (stunting) in the intervention children compared with the controls, nor was there a difference in fat mass or fat distribution (skinfold thickness) (Table 3).

**TABLE 2:** Differences in primary outcomes between intervention and control protocols at 12 and 24 wk post enrollment, disaggregated by risk group and for all children^1^

	High risk only		Low risk only		All enrolled children			
	Intervention (*n* = 317)	Control (*n* = 393)	Intervention (*n* = 256)	Control (*n* = 321)	Intervention (*n* = 573)	Control (*n* = 714)	Adjusted rd (95% CI)^2^	*P* value
12-wk outcomes								
Recovered	134 (42.3)	143 (36.3)	139 (54.3)	135 (42.1)	273 (47.6)	278 (38.9)	0.08 (0.04, 0.13)	<0.001
Died	4 (1.3)	12 (3.1)	6 (2.3)	10 (3.2)	10 (1.8)	22 (3.1)	−0.02 (−0.03, −0.00)	0.042
Deteriorate to SAM by follow-up	78 (24.6)	126 (32.0)	24 (9.4)	52 (16.2)	100 (17.5)	174 (24.4)	−0.07 (−0.11, −0.04)	<0.001
Remained with MAM	89 (28.1)	86 (21.8)	73 (28.5)	109 (34.0)	162 (28.3)	195 (27.3)	−0.01 (−0.06, 0.05)	0.922
Recent illness^3^	76 (24.0)	90 (22.9)	48 (18.8)	80 (24.9)	124 (21.6)	170 (23.8)	−0.04 (−0.09, 0.02)	0.170
24-wk outcomes								
Recovered	115 (36.2)	134 (34.1)	134 (52.3)	136 (42.3)	249 (43.5)	270 (37.8)	0.06 (0.02, 0.11)	0.007
Died	9 (2.8)	20 (5.1)	10 (3.9)	17 (5.3)	19 (3.3)	37 (5.2)	−0.02 (−0.04, 0.00)	0.057
Deteriorate to SAM by follow-up	127 (40.1)	147 (37.4)	45 (17.6)	86 (26.8)	178 (31.1)	244 (34.2)	−0.03 (−0.09, 0.02)	0.241
Remained with MAM	32 (10.1)	30 (7.6)	32 (12.5)	44 (13.7)	64 (11.2)	74 (10.4)	0.01 (−0.04, 0.05)	0.744
Relapsed^4^	16 (5.1)	20 (5.1)	14 (5.5)	24 (7.5)	30 (5.2)	44 (6.2)	−0.01 (−0.04, 0.02)	0.443
Recent illness^3^	50 (18.3)	69 (22.6)	51 (19.9)	69 (21.5)	101 (20.7)	138 (23.8)	−0.04 (−0.10, 0.01)	0.151

^1^Values are *n* (%) or mean (95% CI) unless otherwise indicated. Outcomes were compared using logistic regression analysis (statistical comparison was not conducted for risk subgroups owing to the risk of being underpowered). MAM, moderate acute malnutrition; rd, risk difference; SAM, severe acute malnutrition.

^2^Rd adjusted for age and sex, and model accounted for clustering by clinic site.

^3^Diarrhea, rash, fever, or cough in the past 14 d.

^4^Developed MAM having previously recovered.

**TABLE 3:** Anthropometry at 12 and 24 wk post enrollment^1^

	12 wk post enrollment				24 wk post enrollment			
	Intervention (*n* = 545)	Control (*n* = 665)	Mean difference^2^	*P* value	Intervention (*n* = 534)	Control (*n* = 661)	Mean difference^2^	*P* value
MUAC, cm	12.51 ± 0.81	12.42 ± 0.88	0.08 (−0.02, 0.18)	0.11	12.72 ± 0.98	12.63 ± 1.00	0.09 (−0.03, 0.21)	0.14
WAZ	−2.60 ± 0.91	−2.68 ± 0.98	0.09 (−0.02, 0.19)	0.10	−2.55 ± 1.01	−2.64 ± 1.00	0.10 (−0.02, 0.21)	0.09
LAZ	−2.96 ± 1.11	−2.98 ± 1.20	0.04 (−0.08, 0.16)	0.56	−3.01 ± 1.11	−2.99 ± 1.20	−0.01 (−0.14, 0.12)	0.93
WLZ	−1.42 ± 0.89	−1.52 ± 0.94	0.10 (−0.02, 0.22)	0.10	−1.37 ± 0.99	−1.50 ± 0.97	0.14 (0.02, 0.26)	0.02
Subscapular skinfold-for-age *z*	−0.60 ± 1.29	−0.65 ± 1.45	0.05 (−0.11, 0.21)	0.51	−0.30 ± 1.37	−0.39 ± 1.47	0.10 (−0.08, 0.28)	0.28
Triceps skinfold-for-age *z*	−0.76 ± 1.12	−0.78 ± 1.19	0.02 (−0.11, 0.16)	0.73	−0.47 ± 1.22	−0.51 ± 1.21	0.04 (−0.10, 0.19)	0.55
Skinfold thickness ratio^3^	1.21 ± 0.23	1.21 ± 0.22	−0.01 (−0.03, 0.02)	0.77	1.22 ± 0.24	1.23 ± 0.23	−0.01 (−0.03, 0.02)	0.87
Change in MUAC, cm	0.57 ± 0.77	0.45 ± 0.86	0.12 (0.03, 0.21)	0.01	0.79 ± 0.95	0.66 ± 0.99	0.13 (0.02, 0.24)	0.03
Weight gain, g · kg^−1^ · d^−1^	1.24 ± 0.95	1.10 ± 1.05	0.15 (0.03, 0.26)	0.02	1.07 ± 0.67	0.97 ± 0.64	0.11 (0.03, 0.18)	0.01

^1^Values are means ± SDs or means (95% CIs) unless otherwise indicated. Outcomes were compared using linear regression analysis. LAZ, length-for-age *z* score; MUAC, midupper arm circumference; WAZ, weight-for-age *z* score; WLZ, weight-for-length *z* score.

^2^Mean difference adjusted for age and sex, and model accounted for clustering by clinic site.

^3^Ratio is subscapular:triceps.

Although our analysis was not powered for disaggregated comparison between risk groups, some of the differences between the intervention and control groups at 12 wk were driven by differences in recovery between the 2 low-risk groups (54% compared with 42%, 12% difference), both of which received counseling only (Figure 2). When comparing high-risk children only (Figure 2, Table 2, **Supplemental Table 3**), we found that recovery, survival, and preventing SAM were still significantly better in the intervention group in the short term, especially at 6 wk (45% compared with 24%) when most children had just completed their course of RUTF, but superiority of the intervention over counseling receded by 12 and 24 wk (recovery at 12 wk = 42% compared with 36%, 6% difference).

**FIGURE 3:**
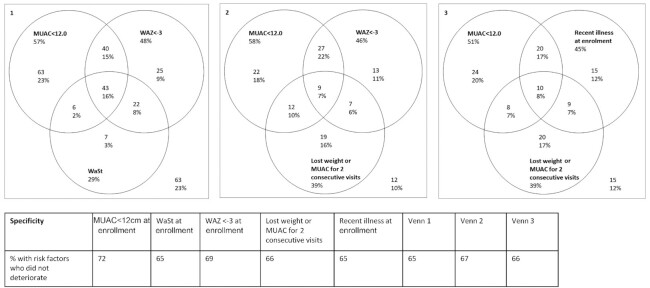
Proportion of children at control sites who died or deteriorated by 24 wk based on risk factors. Three Venn diagrams show the numbers and proportions of children who deteriorated that were identified by various combinations of risk factors. For example, in diagram 1, 57% of children who deteriorated had MUAC <12 cm; 23% had MUAC <12 cm only [i.e., neither of the other 2 factors (WAZ <−3 or WaSt)]. Another 23% had none of these 3 risk factors (number in the bottom right of the square). The table shows the specificity of each risk factor individually and for the combinations of risk factors in each of the Venn diagrams (i.e., the proportion of children without the risk factor and who did not deteriorate). MUAC, midupper arm circumference; WaSt, concurrent wasting and stunting; WAZ, weight-for-age *z* score.

Using the control group data only, we identified risk factors associated with poor outcomes. **Table 4** shows that there was no difference in the odds of deteriorating to SAM or death based on the child's mother not being the caregiver, or breastfeeding status, 2 of our a priori criteria. Lower MUAC and WAZ did show greater odds of a poor outcome at the study control sites. Children who had either a drop in weight or a drop in MUAC for 2 consecutive visits during their participation in mother care support groups were more likely to deteriorate or die by 24 wk. Illness in the previous 2 wk was also associated with a poor outcome—particularly fever and/or diarrhea at enrollment or 6 wk after enrollment. Of those with a poor outcome, 34% had fever at 6 wk compared with 23% of those who did not have a poor outcome (OR: 1.68; 95% CI: 1.11, 2.52); for diarrhea the figures were 6% compared with 2% (OR: 3.80; 95% CI: 1.32, 10.97). Younger age, being a twin, and having had SAM in the past were also associated with deterioration. **Figure 3** presents the sensitivity and specificity of different combinations of risk factors for predicting deterioration. We found that 49% (129 of 261) of those with MUAC < 12 cm at enrollment deteriorated to SAM or died by 24 wk, whereas 31% (141 of 453) deteriorated or died if their MUAC was ≥12 cm (**Supplemental Figure 3**).

**TABLE 4:** Characteristics associated with deterioration or death by 24 wk among control site children only^1^

	Deteriorated or died (*n* = 270)	Did not deteriorate/die (*n* = 444)	Adjusted OR (95% CI); *P* value
Anthropometry			
WAZ <−3.5 at enrollment	55 (20.4)	67 (15.1)	2.13 (1.35, 3.37); 0.001
WAZ <−3.0 at enrollment	130 (48.1)	168 (37.8)	2.01 (1.42, 2.84); <0.001
MUAC <12.0 cm at enrollment	129 (47.8)	132 (29.7)	2.09 (1.52, 2.87); <0.001
WAZ at enrollment	−2.96 ± 0.72	−2.79 ± 0.72	0.50 (0.38, 0.65); <0.001
MUAC at enrollment	11.9 ± 0.3	12.0 ± 0.3	0.22 (0.12, 0.40); <0.001
LAZ at enrollment	−2.95 ± 1.2	−2.72 ± 1.2	0.74 (0.64, 0.86); <0.001
WaSt at enrollment	78 (28.9)	128 (28.8)	1.21 (0.83, 1.75); 0.324
Lost weight or MUAC for 2 consecutive visits	47 (17.4)	29 (6.5)	2.92 (1.77, 4.81); <0.001
Subscapular skinfold-for-age *z* at enrollment	−1.29 ± 1.09	−1.22 ± 1.00	0.94 (0.81, 1.10); 0.45
Triceps skinfold-for-age *z* at enrollment	−1.43 ± 0.90	−1.36 ± 0.87	0.95 (0.80, 1.13); 0.57
Child and mother demographics			
Age, mo	12.2 ± 6.2	13.7 ± 6.9	0.97 (0.94, 0.99); 0.004
Sex (boys)	110 (40.7)	174 (39.2)	1.06 (0.78, 1.45); 0.698
Twin	12 (4.4)	5 (1.1)	3.87 (1.34, 11.17); 0.012
Mother not caregiver	19 (7.0)	46 (10.4)	0.79 (0.44, 1.41); 0.425
<2 y old and not breastfed	30 (11.1)	63 (14.2)	0.91 (0.56, 1.48); 0.70
Caregiver with no education	149 (55.2)	259 (58.3)	0.89 (0.65, 1.21); 0.445
Maternal age, y	25.4 ± 6.5	26.3 ± 8.2	0.99 (0.96, 1.01); 0.288
Health history			
Recent illness before enrollment	122 (45.2)	201 (45.3)	0.99 (0.73, 1.34); 0.940
Recent illness before 6-wk visit	85 (31.5)	80 (18.0)	1.71 (1.17, 2.52); 0.006
Recent illness before 12-wk visit	88 (36.8)	82 (22.0)	2.10 (1.46, 3.02); <0.001
Known treated for MAM in 24 mo before enrollment	54 (20.0)	95 (21.4)	1.16 (0.77, 1.74); 0.479
Ever treated for SAM in the past	88 (32.6)	127 (28.7)	1.43 (1.01, 2.01); 0.044
Food security			
Food security score (FIES)	6.1 ± 2.1	6.1 ± 2.1	0.98 (0.91, 1.05); 0.602

^1^Values are *n* (%) or mean ± SD unless otherwise indicated. Results of logistic regression analysis within control group only, for those who deteriorated to SAM or died within 24 wk. OR adjusted for age and sex, accounting for clusters. FIES, Food Insecurity Experience Scale; LAZ, length-for-age *z* score; MAM, moderate acute malnutrition; MUAC, midupper arm circumference; SAM, severe acute malnutrition; WaSt, concurrent wasting and stunting; WAZ, weight-for-age *z* score.

The average cost of providing RUTF and amoxicillin for an HR-MAM child was USD12.10. The cost of RUTF for SAM treatment for those who deteriorated was USD37/child. The average RUTF expenditure per child treated was USD13 at the intervention sites compared with USD9 at the control sites. Per child recovered, the RUTF costs were similar between the 2 groups: USD27 and USD23 for the intervention and control sites, respectively.

## Discussion

The best strategies for supporting children suffering from MAM need evidence to inform policy and programs, and this study offers initial data to fill that important gap (10). We found that provision of 1 sachet of RUTF per day and amoxicillin to HR-MAM children reduced their risk of a poor outcome while they were receiving treatment (i.e., before 12 wk), and increased MUAC and weight gain, compared with nutrition counseling; however, benefits in recovery and risk of deterioration were not sustained. Our results also reveal that more than one-quarter of children with MAM in this nonemergency context will deteriorate within 3 mo if treated with a counseling intervention alone.

Unfortunately, recovery rates were suboptimal in both groups and a large proportion of children deteriorated to SAM or relapsed to MAM, even among those who were discharged as recovered. Only 42% of the HR-MAM children at the intervention sites recovered by 12 wk without the need for SAM treatment; 28% remained moderately wasted and 25% developed SAM or died. The provision of RUTF and antibiotics also did not provide significantly sustained increases in anthropometry after 24 wk. A recent study of 2683 MAM children in the same district in Sierra Leone that provided supplementary foods to all children with MAM found similarly high rates of deterioration. They found that 63% recovered, 10% remained with MAM, 19% developed SAM, and 1% died within 12 wk (**Supplemental Figure 4**) (25). In combination, these results suggest that providing food (either to all, or to some MAM children in combination with antibiotics) prevents ∼7% points of poor outcomes at 12 wk compared with counseling alone.

A 12-mo follow-up of children with MAM given supplementary food in Malawi also found high rates of deterioration (9). Both of these studies were set in poor, nonemergency settings where MAM is likely to be the consequence of both chronic factors and intermittent insults. A more comprehensive package of interventions, beyond counseling and food supplementation, may help, and/or longer duration of support. Most high-risk children in our study received RUTF for 5 wk; this was until they had MUAC > 12.5 cm for 2 consecutive visits. A study in Malawi provided recent MAM recoverees with a package of health and nutrition interventions, including a lipid nutrient supplement for 8 wk, deworming medication, zinc supplementation, a bed net, and malaria chemoprophylaxis. However, only 56% of intervention children and 53% in the control group sustained recovery after 12 mo (26). Our findings suggest that MAM in a nonemergency context is difficult to successfully treat, most likely because of ongoing insults from the environment, food insecurity, and infectious diseases.

The WHO has questioned whether food supplementation is necessary in nonemergency contexts, and others have suggested it may even increase the risk of excessive weight gain (8, 27). We found no evidence of excessive or unhealthy weight gain, based on skinfold thickness, in those who received RUTF compared with those who did not. The average skinfold thickness *z* scores for both groups remained below the global average—a similar finding to those of a 4-mo follow-up study of MAM children treated with RUTF in Kenya (28). This suggests that the option of food supplementation is likely to be safe for children across the spectrum of acute malnutrition; however, there is still a need to identify and prioritize those at highest risk (11). Food supplementation does not necessarily mean RUTF, because studies have seen similar recovery rates when using other lipid-based nutrient supplements; however, meta-analyses have suggested that fortified blended flours are not as effective (12, 29, 30).

Within the population of children with MAM, further risk stratification can be used to more specifically target supplementary feeding. The definition of “high risk” warrants more precise characterization. Our control group suggests that low MUAC (<12.0 cm), low WAZ (<−3), lower LAZ, dropping anthropometry, reported recent illness (especially fever or diarrhea), younger age (approximately < 12 mo), being a twin, and having a history of previous SAM are significant risk factors for deterioration. A study in Ethiopia of untreated MAM also found MUAC < 12.0 cm to be a key risk factor for deterioration (16). Low WAZ alone can be used to predict concurrent wasting and stunting and has been identified previously as a risk factor for death (18). In hindsight, our a priori defined risk factors were not wholly appropriate. “Mother not the primary caregiver” and “less than 2 years and not breastfeeding” were not associated with risk of deterioration among our control children. Because these risk factors defined 21% of the sample, this may have affected our results by diluting the effect of RUTF and antibiotics on the HR-MAM group. This study has, however, identified a combination of 3 practical risk factors: MUAC < 12.0 cm, WAZ < −3, and declining anthropometry during treatment, which can predict 90% of deteriorations with 67% specificity in this context. The addition of indicators to current programming may, however, compromise the desired simplicity for frontline health workers.

With regard to the LR-MAM groups, another factor which may have affected our results was the difference in adherence to counseling, which was higher in the intervention group than in the control group. Adherence has been a challenge in other nutrition counseling studies (10, 31). Greater defaulting and greater declining to take part in the control group may have also affected these results. We explored other factors that could have boosted the recovery rate in the LR-MAM intervention group; however, baseline maternal education, indicators of socio-economic status, and baseline morbidities were similar between the groups and did not alter the results when included in the regression model.

### Limitations

Our study is limited in generalizability and scalability. The context of the study was one of chronic poverty among subsistence farmers where malaria was endemic. Our finding may not be relevant in urban settings, emergency contexts, Asian contexts, or where infectious diseases are more intermittent. Our mother support groups were bolstered by research staff, so their effectiveness once implemented at scale may differ. Integration with existing health platforms such as community health workers could improve effectiveness at scale. It is also not possible to disaggregate the effects of amoxycillin from those of RUTF in our intervention group. The unexpectedly high recovery rates in the LR-MAM intervention group are difficult to explain; different levels of adherence to treatment between the groups suggest that RUTF may enhance the effect of counseling. More participants also declined to participate in the control group than in the intervention group, which may have introduced some selection bias (9% compared with 5%). Despite the limitations, this exploratory study has raised a number of important questions for future research.

### Conclusions

The necessity of food supplementation for all children with MAM has been a topic of recent debate. Our study adds to the body of literature that MAM is a warning sign of potential deterioration to SAM and increased mortality risk. Our findings suggest that nutrition counseling alone is not sufficient for all children with MAM; however, even with a short course of RUTF and antibiotics, recovery rates were suboptimal and protection against SAM did not last beyond the first 3 mo. A longer or more holistic package of interventions may be necessary. Our results, building on previous studies, support a shift in the current management of acute malnutrition in favor of a model which provides a continuum of care for all acutely malnourished children through better identification of risk. Low MUAC, low WAZ, declining anthropometry, young age, twin status, history of SAM, and recent morbidity are appropriate criteria for defining high-risk in this context. Given the large and growing burden of wasting, and the questions arising from this study, further research and operational trials, in a range of contexts, need to carry these findings forward if we are to prevent and treat the majority of malnourished children and meet Sustainable Development Goal 2.

## Supplementary Material

nqab137_Supplemental_File

## Data Availability

Data described in the article, code book, and analytic code will be made available upon request pending application to MM (manarymj@wustl.edu) and approval.

## Notes

Supported by the innocent foundation (to MM). The funders played no role in the design, implementation, analysis, and interpretation of the data.

MM is an associate editor for *The American Journal of Clinical Nutrition*.

Supplemental Tables 1–3 and Supplemental Figures 1–4 are available from the “Supplementary data” link in the online posting of the article and from the same link in the online table of contents at https://academic.oup.com/ajcn/.

Abbreviations used: HR-MAM, high-risk moderate acute malnutrition; LAZ, length-for-age *z* score; LR-MAM, low-risk moderate acute malnutrition; MAM, moderate acute malnutrition; MUAC, midupper arm circumference; rd, risk difference; RUTF, ready-to-use therapeutic food; SAM, severe acute malnutrition; SFP, supplementary feeding program; WLZ, weight-for-length *z* score.
