# Supplementary material for: Treating high-risk moderate acute malnutrition using therapeutic food compared with nutrition counseling (Hi-MAM Study): a cluster-randomized controlled trial
Source: Am J Clin Nutr. 2021 May 8;114(3):955–64. doi: 10.1093/ajcn/nqab137 (PMC8921644; doi:10.1093/ajcn/nqab137)
Supplement: nqab137_Supplemental_File [file nqab137_supplemental_file.docx]

**On-line Supplementary Material**

# *Lelijveld et al., Treating high-risk moderate acute malnutrition using therapeutic food compared to nutrition counselling (Hi-MAM Study): A cluster-randomized controlled trial*

**Supplementary Table 1: sample size by risk group and clinic site**

| **Clinic sites** | **N^o^ of high-risk children in analysis** | **N^o^ of low-risk children in analysis** | **Ratio high risk to low risk** |
| --- | --- | --- | --- |
| **Controls sites** | | |  |
| **A** | **38** | **25** | **1.52** |
| **B** | **53** | **53** | **1.00** |
| **C** | **31** | **17** | **1.82** |
| **D** | **18** | **17** | **1.06** |
| **E** | **19** | **20** | **0.95** |
| **F** | **63** | **37** | **1.70** |
| **G** | **49** | **42** | **1.17** |
| **H** | **41** | **44** | **0.93** |
| **I** | **21** | **19** | **1.11** |
| **J** | **18** | **17** | **1.06** |
| **K** | **42** | **30** | **1.40** |
| **Intervention sites** | | |  |
| **L** | **23** | **19** | **1.21** |
| **M** | **37** | **26** | **1.42** |
| **N** | **41** | **38** | **1.08** |
| **O** | **50** | **44** | **1.14** |
| **P** | **34** | **28** | **1.21** |
| **Q** | **13** | **13** | **1.00** |
| **R** | **33** | **32** | **1.03** |
| **S** | **20** | **17** | **1.18** |
| **T** | **20** | **17** | **1.18** |
| **U** | **29** | **16** | **1.81** |
| **V** | **17** | **6** | **2.83** |

**Comparison of the ratios of SAM: MAM children between intervention and control clinics using Mann-Whitney Test. Median for intervention site is 1.18, for controls 1.11 U=45, P=0.41**

**Supplementary Table 2: extended table of baseline socio-economic categorical characteristics, with statistical comparison between intervention and control groups**

|  | Intervention N=11 sites, n=573 | | | Control N=11 sites, n=714 | | | Control vs intervention Coefficientǂ  (95% CI)  P value |
| --- | --- | --- | --- | --- | --- | --- | --- |
|  | **All** | **High risk n=317 (55.3%)** | **Low risk**  **n=256 (44.7%)** | **All** | **High risk**  **n=393 (55.0%)** | **Low risk**  **n=321 (45.0%)** |  |
| Caregiver educational level | |  |  |  |  |  |  |
| None, n (%) | 334 (58.3) | 189 (59.6) | 145 (56.6) | 408 (57.5) | 204 (51.9) | 204 (64.4) | 0.05  (-0.18, 0.27)  0.68 |
| Primary, n (%) | 116 (20.2) | 55 (17.4) | 61 (23.8) | 121 (17.4) | 68 (17.3) | 53 (16.7) | 0.22  (-0.06, 0.50)  0.13 |
| >primary, n (%) | 123 (21.5) | 73 (23.0) | 50 (19.5) | 181 (25.0) | 121 (30.7) | 60 (18.9) | -0.22  (-0.49, 0.04)  0.09 |
| Food insecurity experience scale, n (%): | | |  |  |  |  |  |
| Least insecure (score 0) | 27 (4.8) | 17 (5.4) | 10 (3.9) | 43 (6.1) | 28 (7.2) | 15 (4.7) | -0.26  (-0.75, 0.24)  0.30 |
| Most insecure (score 8) | 126 (22.2) | 60 (19.1) | 66 (25.9) | 149 (21.1) | 71 (18.3) | 78 (24.6) | 0.07  (-0.20, 0.33)  0.63 |
| Animals sleep in house,  n (%) | 93 (16.2) | 46 (14.5) | 47 (18.4) | 127 (17.9) | 75 (19.1) | 52 (16.4) | -0.12  (-0.41, 0.18)  0.44 |
| Metal sheet roof vs thatched (n, %) | 437  (76.3) | 246  (77.6) | 191  (74.6) | 570 (79.8) | 318  (80.9) | 252  (78.5) | -0.21  (-0.47, 0.06)  0.12 |
| Access to a mobile phone (n, %) | 324 (56.5) | 182 (57.4) | 142 (55.5) | 459 (64.6) | 268 (68.4) | 191 (59.9) | -0.34  (-0.56, -0.11)  0.003 |
| Household owns a motorbike | 121 (22.2) | 64 (21.0) | 57 (23.8) | 166 (24.2) | 99 (25.9) | 67 (22.0) | -0.11  (-0.28, 0.16)  0.42 |
| Improved toilet facilities* | 371 (64.8) | 211 (66.6) | 160 (62.5) | 433 (60.6) | 244 (62.1) | 189 (58.9) | 0.18  (-0.05, 0.40)  0.13 |
| Child ever treated for SAM, n (%) | 182 (31.8) | 100 (31.6) | 82 (32.0) | 215 (30.2) | 139 (35.3) | 76 (23.9) | 0.07  (-0.17, 0.31)  0.57 |
| Child ever admitted to hospital, n (%) | 77 (13.4) | 41 (12.9) | 36 (14.1) | 78 (11.0) | 46 (11.7) | 32 (10.1) | 0.07  (-0.17, 0.31)  0.57 |

*improvement on no facility or shared open pit ǂunadjusted logistic regression

**Supplementary Table 3: Continuous outcomes at 12 and 24 for intervention and control groups, disaggregated by low- and high-risk designation**

|  | **At 12 weeks post-enrolment** | | | | **At 24 weeks post-enrolment** | | | |
| --- | --- | --- | --- | --- | --- | --- | --- | --- |
|  | **Intervention,**  Mean (SD) | | **Control,**  Mean (SD) | | **Intervention**,  Mean (SD) | | **Control,**  Mean (SD) | |
|  | High risk | Low risk | High risk | Low risk | High risk | Low risk | High risk | Low risk |
| MUAC (cm) | 12.39 (0.86) | 12.65 (0.72) | 12.38 (0.88) | 12.47 (0.87) | 12.67 (1.00) | 12.79 (0.95) | 12.60 (1.04) | 12.67 (0.96) |
| WAZ | -2.78 (0.96) | -2.37 (0.78) | -2.88 (1.02) | -2.44 (0.89) | -2.72 (1.08) | -2.32 (0.86) | -2.86 (1.02) | -2.39 (0.93) |
| LAZ | -3.12 (1.18) | -2.75 (0.95) | -3.28 (1.28) | -2.63 (1.00) | -3.20 (1.21) | -2.77 (0.91) | -3.31 (1.26) | -2.64 (1.02) |
| WLZ | -1.54 (0.92) | -1.26 (0.81) | -1.56 (0.93) | -1.47 (0.97) | -1.46 (1.03) | -1.26 (0.90) | -1.55 (0.98) | -1.44 (0.96) |
| Subscapular skinfold-for-age Z | -0.74 (1.34) | -0.40 (1.18) | -0.50 (1.49) | -0.82 (1.38) | -0.34 (1.36) | -0.25 (1.39) | -0.23 (1.50) | -0.58 (1.42) |
| Tricep skinfold-for-age Z | -0.85 (1.14) | -0.63 (1.08) | -0.73 (1.24) | -0.84 (1.11) | -0.47 (1.25) | -0.48 (1.18) | -0.44 (1.23) | -0.59 (1.17) |
| Skinfold thickness ratio ~ | 1.22 (0.24) | 1.19 (0.22) | 1.20 (0.23) | 1.23 (0.22) | 1.24 (0.24) | 1.20 (0.23) | 1.22 (0.23) | 1.24 (0.22) |
| Change in MUAC (cm) | 0.61 (0.81) | 0.52 (0.71) | 0.56 (0.84) | 0.32 (0.87) | 0.88 (0.97) | 0.66 (0.92) | 0.78 (0.99) | 0.52 (0.96) |
| Average weight gain (g/kg/day) | 1.29 (0.98) | 1.18 (0.89) | 1.16 (1.05) | 1.03 (1.05) | 1.10 (0.68) | 1.04 (0.65) | 0.98 (0.64) | 0.95 (0.64) |

*relapse = developed MAM having previously recovered. ^$^diarrhoea, rash, fever or cough in the past 14 days. SAM= severe acute malnutrition. MUAC=mid-upper arm circumference. MAM= moderate acute malnutrition. ~ ratio is subscapular/tricep. WAZ= weight for age z-score; LAZ= length for age z-score; WLZ= weight for length z-score.

**Supplementary Figure 1: Kaplan Meier curve showing the difference in deterioration to SAM or death between the two study arms**


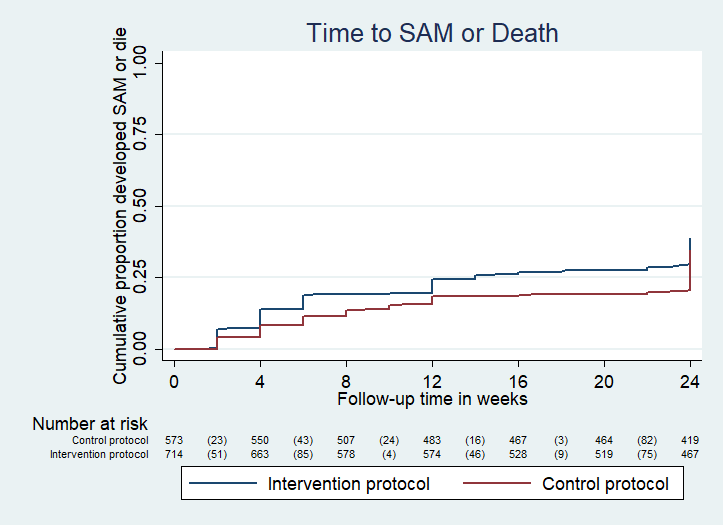


**Supplementary Figure 2: MUAC gains since enrolment for the 4 study groups.**


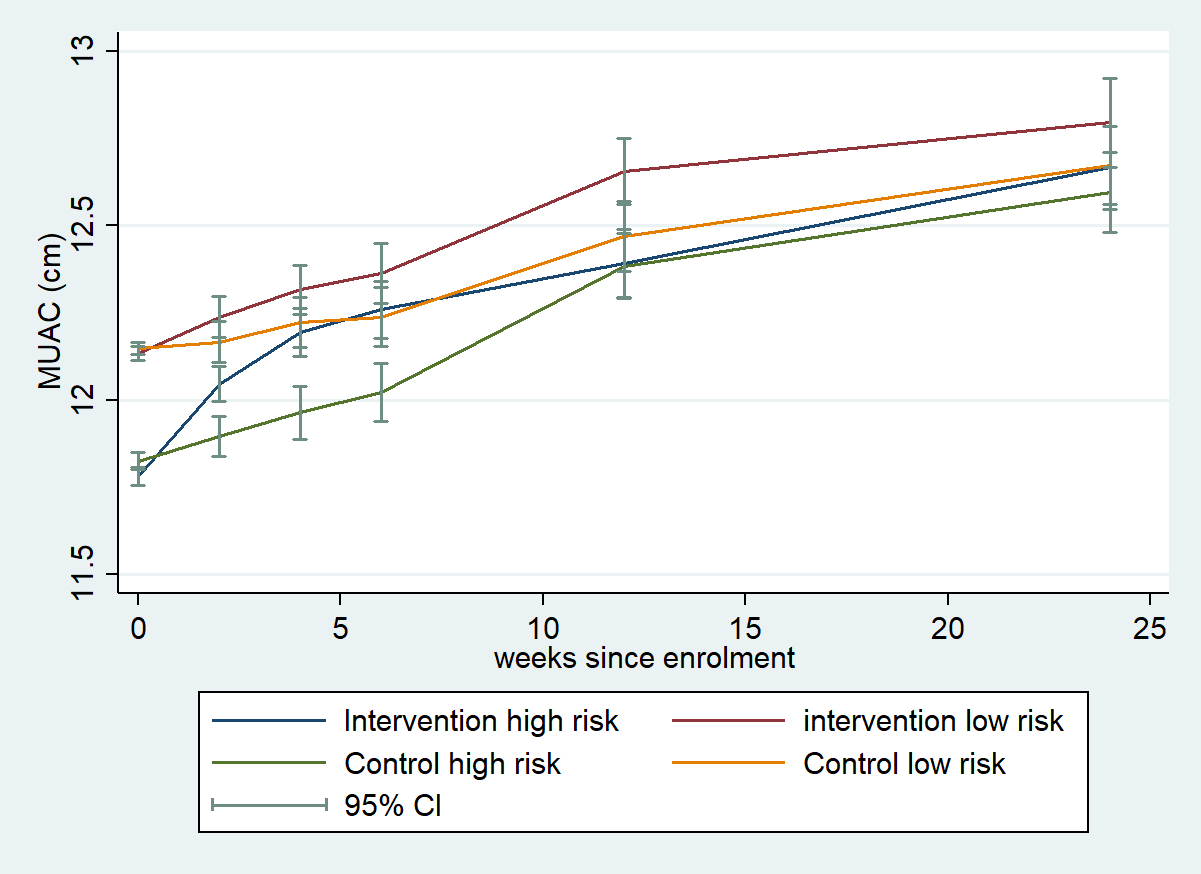


*NB. This graph also includes children who received SAM treatment.

**Supplementary Figure 3: difference in deterioration in the control group for those with MUAC < 12 cm and MUAC ≥ 12cm.**

**
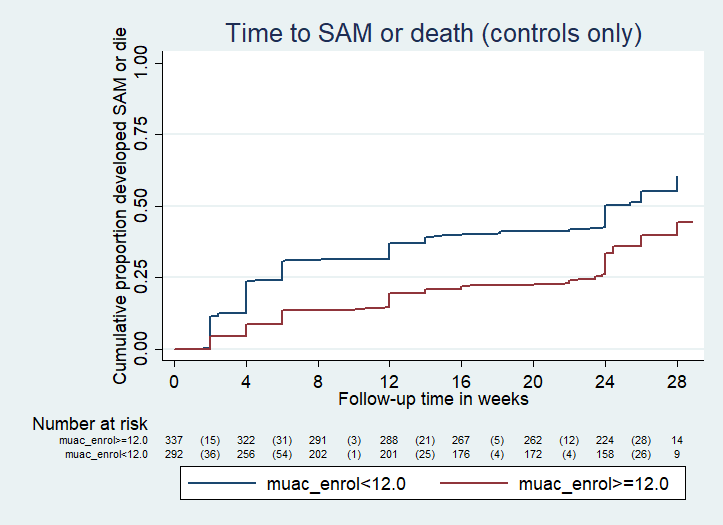
**

**Supplementary Figure 4**: Comparison of intervention and control protocol outcomes with those of the “Four Foods” study that provided supplementary food to all MAM children in Pujehun District in Sierra Leone [23]*

* Langlois, B., et al., *Comparative Effectiveness of Four Specialized Nutritious Food Products for Treatment of Moderate Acute Malnutrition in Sierra Leone (P10-140-19).* Current developments in nutrition, 2019. **3**(Supplement_1): p. nzz034. P10-140-19.
